# Accuracy and prognostic impact of FDG PET/CT and biopsy in bone marrow assessment of follicular lymphoma at diagnosis: A Nation‐Wide cohort study

**DOI:** 10.1002/cam4.5424

**Published:** 2022-11-13

**Authors:** Isabel Ródenas‐Quiñonero, Tzu Chen‐Liang, Taida Martín‐Santos, Antonio Salar, Marta Fernández‐González, Carolina Celades, José‐Tomás Navarro, Ana Belén Martínez‐Garcia, Rafael Andreu, Aitana Balaguer, Alejandro Martin García‐Sancho, Mónica Baile, Javier López‐Jiménez, Juan Marquet‐Palomanes, Ana Isabel Teruel, María José Terol, Carmen Benet, Laura Frutos, José Luis Navarro, Jon Uña, Marina Suarez, Montserrat Cortes, José Contreras, Cristina Ruiz, Pilar Tamayo, Jorge Mucientes, Pablo Sopena‐Novales, Laura Reguilón‐Gallego, José Javier Sánchez‐Blanco, Elena Pérez‐Ceballos, Andrés Jerez, Francisco José Ortuño

**Affiliations:** ^1^ S. de Hematología y Oncología Médica H.J.M. Morales Meseguer, IMIB‐Pascual Parrilla. Murcia Spain; ^2^ S. de Hematología H. Universitario de Canarias La Laguna, Tenerife Spain; ^3^ S. de Hematología H. del Mar Barcelona Spain; ^4^ S. de Hematología Josep Carreras Leukaemia Research Institute (IJC) Badalona Spain; ^5^ S. de Hematología ICO‐H. Germans Trias i Pujol Badalona Spain; ^6^ S. de Hematología H. Sta. Lucia Cartagena Spain; ^7^ S. de Hematología H. La Fe Valencia Spain; ^8^ S. de Hematología H. Clínico Universitario, Salamanca, IBSAL, CIBERONC Valladolid Spain; ^9^ S. de Hematología H. Ramón y Cajal Madrid Spain; ^10^ S. de Hematología H. Clínico Valencia Spain; ^11^ S. de Hematología H. Arnau de ViIlanova Valencia Spain; ^12^ S. de Medicina Nuclear H. Virgen de la Arrixaca Murcia Spain; ^13^ S. de Medicina Nuclear H. Universitario N.S. de la Candelaria Tenerife Spain; ^14^ S. de Medicina Nuclear H. del Mar Barcelona Spain; ^15^ S. de Medicina Nuclear H. Universitari de Bellvitge‐IDIBELL Barcelona Spain; ^16^ S. de Medicina Nuclear H. Santa Lucia Cartagena, Murcia Spain; ^17^ S. de Medicina Nuclear H. La Fe Valencia Spain; ^18^ S. de Medicina Nuclear H. Clínico Universitario de Salamanca/IBSAL Salamanca Spain; ^19^ S. de Medicina Nuclear H. Puerta de Hierro Madrid Spain; ^20^ S. de Medicina Nuclear H. 9 de Octubre Valencia Spain

**Keywords:** bone marrow biopsy, follicular lymphoma, PET/CT

## Abstract

**Backgound:**

In the workup of follicular lymphoma (FL), bone marrow biopsy (BMB) assessment is a key component of FLIPI and FLIPI2, the most widely used outcome scores. During the previous decade, several studies explored the role of FDG‐PET/CT for detecting nodal and extranodal disease, with only one large study comparing both techniques.

**Methods:**

The aim of our study was to evaluate the diagnostic accuracy and the prognostic impact of both procedures in a retrospective cohort of 299 FL patients with both tests performed at diagnosis. In order to avoid a collinearity bias, FLIPI2 was deconstructed in its founding parameters, and the bone marrow involvement (BMI) parameter separately included as: a positive BMB, a positive PET/CT, the combined “PET/CT and BMB positive” or “PET/CT or BMB positive”. These variables were also confronted independently with the POD24 in 233 patients treated with intensive regimens.

**Results:**

In the total cohort, bone marrow was involved in 124 and 60 patients by BMB and PET/CT, respectively. In terms of overall survival, age > 60 y.o. and the combined “PET/CT or BMB positive” achieved statistical independence as a prognostic factor. In patients treated with an intensive regimen, only the combined “PET/CT or BMB positive” added prognostic value for a shorter overall survival, when confronted with the POD24.

**Conclusion:**

Our results show that in FL both BMB and PET/CT should be considered at diagnosis, as their combined assessment provides independent prognostic value in the context of the most widely use clinical scores.

## 1 INTRODUCTION

In the setting of follicular lymphoma (FL), bone marrow involvement (BMI) is frequently observed and its assessment in the upfront staging is relevant as it might influence patient management.^1^, ^2^, ^3^, ^4^, ^5^, ^6^, ^7^, ^8^ Until recently, bone marrow biopsy (BMB) has been undoubtedly the gold standard for this purpose and is a key variable included in the FLIPI, FLIPI2 and PRIMA‐PI indexes.^5^, ^6^, ^7^ However, a role for positron emission tomography (PET) and PET/computed tomography (PET/CT) in this framework has been proposed by recent studies.^9^, ^10^, ^11^

PET and PET/CT have become essential tools for the management of lymphoma. The outstanding role of PET/CT in Hodgkin lymphoma (HL) has been extensively proved.^12^, ^13^ Regarding diffuse large B‐cell lymphoma (DLBCL), though PET/CT is recommended for staging and evaluation of response,^14^, ^15^, ^16^ it has some potential limitations, particularly concerning BMI analysis.^17^, ^18^ However, its value in FL is more actively discussed: While the 2015 ESMO clinical guidelines favored the routine use of PET/CT in the initial staging,^19^ others did not make it mandatory.^20^

In the FL setting, 18F‐fluorodeoxyglucose (18FDG) nodal avidity has been demonstrated in >95% of cases.^21^, ^22^ Even more, both the PET/CT after induction therapy and the gradient in 18FDG nodal uptake between initial staging and response evaluation have been related with outcome.^23^, ^24^, ^25^, ^26^ In contrast, a number of studies have reported an uneven role of this procedure for routine bone marrow (BM) pretreatment staging,^10^, ^11^, ^27^, ^28^, ^29^ with some groups pointing out a different 18FDG uptake profile between lymph nodes and BM.^30^ To this regard, a wide range of sensitivities have been reported for the assessment of bone marrow infiltration using PET or PET/CT in FL.^10^, ^11^, ^21^, ^25^, ^28^, ^29^, ^30^, ^31^

With the aim of further evaluating the role of both PET/CT and BMB in detecting BMI in the upfront workup of FL, we have recruited a nation‐wide cohort of 299 patients. We have focused on analyzing the accuracy of both tests in the evaluation of BMI in the initial staging and the impact of both procedures on prognosis, with an emphasis to avoid collinearity biases in the multivariate models.

## 2 PATIENTS AND METHODS

### 2.1 Patients

Patients ≥18 years old (y.o.) with a diagnosis of FL grade 1 to 3a according to the WHO classification, between June 2005 and December 2018, with both a BMB and PET/CT performed at baseline were included, from 10 tertiary centers of Spain. Patients had not received either chemotherapy or corticosteroids, and no concomitant malignancy was known to be present at the time of both procedures. Pathology and PET/CT results were unknown to each other specialist.

This study was approved by the University Hospital Morales Meseguer IRB (EST:05/18) and performed in accordance with the Declaration of Helsinki.

### 2.2 Bone marrow biopsy

In Spain, unguided unilateral posterior iliac crest biopsy and marrow aspirate are recommended in patients diagnosed with non‐Hodgkin lymphoma (NHL) according to GELTAMO guidelines, though there is no consensus for FL. Following National Pathology Guidelines, CD20 and CD3 were used to confirm B‐infiltration and rule out reactive mixed nodules.

BMBs were evaluated by experienced hematopathologists in each center. Results were obtained from the individual reports and were not reviewed thereafter. Data from bone marrow aspirate, and either flow cytometry or molecular tests were not used in the present work.

### 2.3 PET/CT imaging and analysis

PET/CT studies were obtained by the following PET/CT devices: Gemini TF64, Gemini GXL and Gemini TF16 (Gemini devices from Philips), Discovery LS, Discovery ST, Discovery STE and Discovery IQ (Discovery devices from GE Healthcare), and either Biograph mCT 20 Flow, Biograph TP16 and Biograph 6 (Biograph devices from Siemens). Procedure, quality control and interpretation guidelines are commented in detail in our previous works.^13^, ^17^ BMI by PET/CT was considered positive with the presence of unifocal (single lesion), bifocal, multifocal (≥3 lesions) or focal lesions with diffuse uptake. Purely diffuse FDG uptake was not considered BMI.

### 2.4 Statistics

We used the Kaplan–Meier and the Cox method to analyze overall survival (OS) and progression free survival (PFS), with a two‐sided *p* value ≤0.15 for a factor in the univariate analysis to be included in the multivariate regression, where a *p* value ≤0.05 was considered statistically significant. To avoid collinearity in the multivariate regressions, we deconstructed the FLIPI2 composite in its foundational factors, considering BMI by mean of different measures in four models: BMB+, PET/CT+, “BMB+ and PET/CT+” and “BMB+ or PET/CT+”. In Cox regressions, examination of log (−log) survival plots and partial residuals was performed to assess that the underlying assumption of proportional hazards was met.

In addition, we tested whether the defined BMI variables added independent prognostic value to the POD24, regarding OS, in the subset of patients intensively treated (immunochemotherapy regimens aimed for remission) in first line. In this group we included either patients treated with Rituximab, Cyclophosphamide, Doxorubicin, Vincristine, Prednisone (R‐CHOP), Rituximab‐Bendamustine (R‐B) and Rituximab, Cyclophosphamide, Vincristine and Prednisone (R‐CVP) and those treated with different high intensity regimens including a number of distinct clinical trials (Table 1).

**TABLE 1:** Patient characteristics

Characteristic	Total cohort (*n* = 299)
Age, median (IQR), y.o.	59 (49–68)
Gender, Female/male, *n* (%)	150 (50.2)/149 (49.8)
Ann Arbor Stage at diagnosis, *n* (%)	
I	28 (9.4)
II	37 (12.4)
III	73 (24.4)
IV	161 (53.8)
FLIPI, *n* (%)	
Low	90 (30.1)
Intermediate	108 (36.1)
High	101 (33.8)
FLIPI2, *n* (%)	
Low	50 (16.7)
Intermediate	159 (53.2)
High	90 (30.1)
Grade, *n* (%)	
I–II	214 (71.5)
III	85 (28.4)
Treatment, *n* (%)	
Rituximab‐CHOP	152 (50.8)
Rituximab‐Bendamustina	37 (12.4)
Rituximab/Obinutuzumab‐CVP	21 (7.0)
Observation, Rituximab, Radiation	53 (17.7)
Others^b^	36 (12.0)
Time to first treatment^a^, median (range), months	1.1 (0–57)
Follow‐up, median (range), months	57.3 (3.6–185.8)

Abbreviations: CHOP, cyclophosphamide, doxorubicin, vincristine, and prednisone; CVP, cyclophosphamide, vincristine, and prednisone; y.o, years old; FLIPI, Follicular Lymphomas International Prognostic Index.

^a^ 287 treated patients.

^b^ Including 23 out of 36 patients treated with high intensity schedules distinct from R‐CHOP, R‐B, R/O‐CVP or/and enrolled in clinical trials.

Accuracy of tests was assessed as previously described.^13^ We used a combined positivity “PET‐CT and BMB” as our gold standard. Statistical analysis was performed using SPSS software (IBM SPSS Statistics 21, IBM Corporation, Chicago, IL) and Epidat (http://dxsp.sergas.es).

## 3 RESULTS

### 3.1 Patient characteristics

A total of 299 FL patients were included. Main characteristics at baseline are shown in Table 1. With a median age at diagnosis of 59 y.o. (interquartile range, 49–68), and a balanced gender distribution (150 females/149 males), the majority of patients (53.8%) had Ann Arbor stage IV. Ninety (30.1%) and 50 (16.7%) patients were included in the low risk category of FLIPI and FLIPI2 indexes, respectively. Most patients had histological grade 1–2 disease (71.5%). Two hundred and thirty‐three patients were treated upfront with immunochemotherapy.

### 3.2 Performance of PET‐CT and BMB findings on staging

The PET/CT was positive for BMI in 58 patients and negative in 241. Among those positive, 37 had also a positive BMB, whereas 87 patients had a positive BMB with a negative PET/CT. The BMB was positive in 124 patients and negative in 175. Among those negative, 21 had a positive PET/CT (Table S1, Appendix S1).

Focusing on the performance by PET/CT, the sensitivity was 40% (95% confidence interval [CI]; 31.6–48.3), negative predictive value (NPV) was 63.9% (95% CI; 57.6–70.1) and accuracy 70.9% (95% CI: 65.5–76.2). Regarding BMB, the sensitivity was 85.5% (95% CI; 79.4–91.5), NPV was 88% (95% CI; 82.9–93.1) and accuracy was 92.8% (95% CI: 89.9–96.0) (Table S2, Appendix S1). Considering BMB as gold standard, the use of PET/CT upstaged 11 patients (3.7%) to Ann Arbor IV. On the other hand, should we considered PET/CT as gold standard, BMB would have upstaged 65 patients (21.7%) to Ann Arbor IV.

We next wanted to check whether those cases with high grade histology behave in a different manner. Among 85 grade 3a FL patients, the PET/CT was positive in 13 patients and negative in 72. Among those positive, 7 also had a positive BMB, whereas 29 patients had a positive BMB with a negative PET/CT. The BMB was positive in 36 patients and negative in 49. Among those negative, 6 had a positive PET/CT (Table S3, Appendix S1). Focusing on the performance by PET/CT, the sensitivity was 26.5% (95% confidence interval (CI; 13.1–39.9)), NPV was 50% (95% CI; 37.7–62.2), accuracy 57.6% (95% CI: 46.5–68.7). Regarding BMB, the sensitivity was 73.4% (95% CI; 60.0–86.8), NPV was 73.4% (95% CI; 60.0–86.8) and accuracy was 84.7% (95% CI: 76.4–92.9) (Table S4, Appendix S1).

### 3.3 Impact of PET‐CT and BMB findings on survival

#### 3.3.1 Whole cohort: deconstructed FLIPI2

**FIGURE 1:**
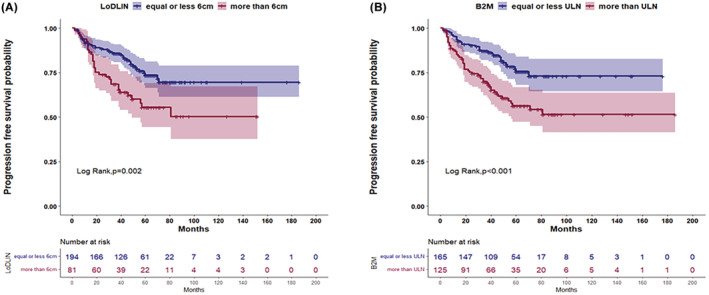
Kaplan–Meier estimate curves of the two factors, within the FLIPI2 score, that remained significant in the multivariate Cox model for PFS. (A) LoDLIN over 6 cm, (B) B2M higher than ULN. B2M, B2‐microglobulin; FLIPI, Follicular Lymphomas International Prognostic Index; LoDLIN, longest diameter of the largest involved nodes; PFS, Progression‐free survival; ULN, upper limit of normal.

With a median (range) follow‐up of 57 months (3–185), 85 patients (28.4%) progressed and 39 (13%) died. Univariate analysis of OS and PFS is shown in Table S5, Appendix S1. A beta2‐microglobulin over the upper normal limit (ULN), a diameter of the largest involved node (LoDLIN) exceeding 6 cm and a hemoglobin lower than 120 g/L, were significantly associated with a shorter PFS in the univariate analysis. From the four different definitions of BMI, a positive BMB and the combined “PET/CT or BMB positive”, were significantly associated with a shorter PFS in univariate analysis. Two multivariate models were created (one for each of the two BMI significant measures) (Table 2). Neither BMI positive or the combined “PET/CT or BMB positive”, could add an independent prognostic value to the two factors than remained significant: beta2‐microglobulin higher than ULN and a LoDLIN over 6 cm (Figure 1).

**TABLE 2:** Prognostic value for PFS and OS of variables considered within the FLIPI2 score (whole cohort). Two multivariate models were performed for PFS. Three multivariate models were performed for OS (Cox proportional hazards model, *n* = 299)

	Multivariate model considering BMB for BMI				Multivariate model considering combined “PET‐CT or BMB” for BMI				Multivariate model considering PET‐CT for BMI	
	PFS p	HR (95% CI)	OS p	HR (95% CI)	PFS p	HR (95% CI)	OS p	HR (95% CI)	OS p	HR (95% CI)
B2M higher than ULN	0.033	1.742 (1.046–2.903)	0.302	1.521 (0.686–3.376)	0.048	1.684 (1.004–2.825)	0.293	1.533 (0.692–3.400)	0.214	1.652 (0.749–3.646)
LoDLIN >6 cm	0.020	1.175 (1.096–2.874)	0.336	1.448 (0.681–3.077)	0.030	1.172 (1.055–2.799)	0.278	1.501 (0.721–3.126)	0.268	1.520 (0.725–3.187)
Hb lower than 120 g/L	0.452	1.248 (0.701–2.221)	0.289	1.582 (0.678–3.693)	0.366	1.302 (0.735–2.307)	0.325	1.513 (0.663–3.450)	0.352	1.485 (0.646–3.413)
Age older than 60 y.o.	0.559	0.868 (0.540–1.396)	0.030	2.359 (1.088–5.113)	0.534	0.860 (0.534–1.384)	0.020	2.473 (1.151–5.314)	0.046	2.180 (1.016–4.680)
BMI by:	NE	NE	NE	NE	NE	NE	NE	NE	0.404	1.388 (0.642–3.000)
PET/TC	0.106	1.479 (0.920–2.378)	0.058	2.024 (0.977–4.190)	NE	NE	NE	NE	NE	NE
BMB	NE	NE	NE	NE	NE	NE	NE	NE	NE	NE
Combined “PET/CT and BMB positive” Combined “PET/CT or BMB positive”	NE	NE	NE	NE	0.095	1.513 (0.931–2.458)	0.030	2.318 (1.083–4.961)	NE	NE

Abbreviations: B2M, B2‐microglobulin; BMB, bone marrow biopsy; BMI, bone marrow involvement; Hb, hemoglobin; HR, hazard ratio; LoDLIN, longest diameter of the largest involved node; NE, not entered; OS, overall survival; PET/CT, PET/computed tomography; PFS, progression‐free survival; ULN, upper limit of normal; y.o, years.

**FIGURE 2:**
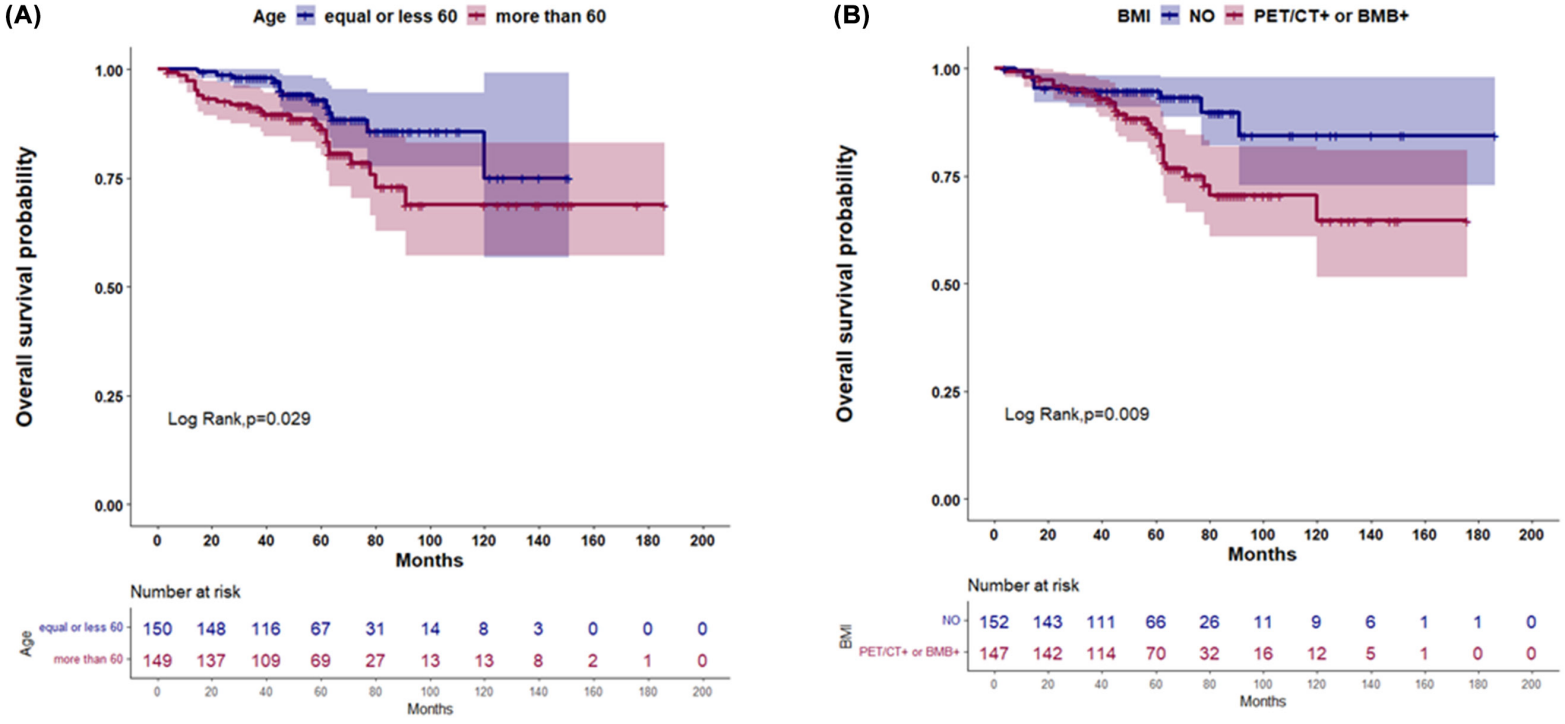
Kaplan–Meier estimate curves of the two factors, within the FLIPI2 score, that remained significant in the multivariate Cox model for OS. (A) Age older than 60 y.o., (B) The combined “PET/CT or BMB positive”. BMB, bone marrow biopsy; BMI, Bone marrow involvement; FLIPI, Follicular Lymphomas International Prognostic Index; OS, overall survival; PET/CT, PET/computed tomography; y.o, years old.

Regarding OS, an elevated beta2‐microglobulin, a LoDLIN over 6 cm, a hemoglobin lower than 120 g/L and an age older than 60 y.o., were significantly associated with a shorter OS in the univariate analysis. Of the four different definitions of BMI, a positive result by BMB, a positive PET/CT and the combined “PET/CT or BMB positive” result, were significantly associated with a shorter OS in univariate regression. Three multivariate models were created (one for each of the two BMI significant measures) (Table 2). The combined “PET/CT or BMB positive” and an age older than 60 y.o. remained significant for a shorter OS (Figure 2).

### 3.4 Intensively treated FL cohort: FLIPI2 and PRIMA‐PI

#### 3.4.1 Deconstructed FLIPI2

We analyzed the cohort of 233 patients treated with immunochemotherapy. Each of the non‐BMI factors of the FLIPI2 and the four different definitions of BMI were tested in univariate analysis for OS and PFS (Table S6, Appendix S1). In the univariate model, a beta2‐microglobulin above ULN, a LoDLIN larger than 6 cm, a hemoglobin lower than 120 g/L, a positive BMB and the combined “PET/CT or BMB positive”, were significantly associated with a shorter PFS. Two multivariate models were created (Table S7, Appendix S1). A LoDLIN over 6 cm, a positive BMB result and the combined “PET/CT or BMB positive”, remained significant for a shorter PFS.

Regarding OS, an elevated beta2‐microglobulin, a LoDLIN over 6 cm, a hemoglobin lower than 120 g/L, a positive BMB, a positive PET/CT and the combined “PET/CT or BMB positive” were significantly associated with a shorter OS in the univariate analysis. Three multivariate models were created (Table S7, Appendix S1). A positive BMB and the combined “PET/CT or BMB positive”, remained significant for a shorter OS.

#### 3.4.2 Deconstructed PRIMA‐PI

PRIMA‐PI score was useful for predicting PFS in patients receiving immunochemotherapy. It involves three groups, the one with the highest risk defined, exclusively, by a beta2‐microglobulin over 3 mg/L. The intermediate and low risk groups required two factors: a beta2‐microglobulin ≤3 mg/L and/or the presence or absence of BMI by BMB, respectively.

We confirmed the prognostic value of presenting a beta2‐microglobulin above 3 mg/L in our series of patients treated with an intensive regimen (*n* = 233) (*p* < 0.01, HR 2.59 (95% CI 1.56–4.30)).

In order to determine which of the four different definitions of BMI provided the best predictive value in PRIMA‐PI score, we selected 171 patients who belonged to the intermediate and low risk groups of this score (those in which BMI is included as a variable). The results of each of the univariate analysis are shown in Table S8, Appendix S1. Only the combined “PET/CT or BMB positive” achieved a statistical significance for predicting PFS.

### 3.5 Intensively treated FL cohort: POD24

In the subset of 233 patients treated with an intensive regimen, we tested whether each of the four variables that we had previously defined for BMI could retain independent prognostic value when confronted with the POD24. Table 3 shows how the only bivariate model in which both the POD24 and a BMI variable kept a significant value was when using the combined “PET/CT or BMB positive”. Though the relative risk of POD24 was 5.4, the presence of a positive result of the combined “PET/CT or BMB positive” represented a three‐fold increased risk of death independently from POD24.

**TABLE 3:** Bivariate models confronting POD24 with the four defined BMI variables (Cox proportional hazards model, *n* = 233)

	Multivariate model	
	OS (p)	HR (95% CI)
PET/CT+	0.230	1.573 (0.571–3.296)
POD24	<0.001	6.302 (3.096–12.827)
BMB+	0.079	2.102 (0.917–4.820)
POD24	<0.001	5.888 (2.809–12.344)
Combined “PET/CT+ and BMB+”	0.463	1.376 (0.587–3.226)
POD24	<0.001	6.300 (3.082–12.878)
Combined “PET/CT+ or BMB+”	0.033	2.887 (1.092–7.633)
POD24	<0.001	5.427 (2.644–11.138)

Abbreviations: BMB, bone marrow biopsy; BMI, bone marrow involvement; HR, Hazard ratio; OS, overall survival; PET/CT, PET/computed tomography; PFS, Progression‐free survival.

## 4 DISCUSSION

We present the largest series so far comparing BMB and PET/CT in the initial staging of FL. Our results show a greater accuracy for BMB over PET/CT both in the whole series and when only 3A histologic grade was considered. Regarding prognosis our data found that the combination “PET/CT or BMB” was the only BMI parameter who kept either a prognostic independent value when considering deconstructed FLIPI‐2 and PRIMA‐PI, or progression considered out with POD24.

During the last decade, a plethora of small series addressed the value of FDG‐PET/CT in FL. In addition, a number of reviews summarized previous reports and focused on both the superiority of the two procedures over conventional CT in detecting nodal and eventually extra nodal disease, and the added value of response assessment through successive studies. Interestingly, those reviews put also into the light a number of methodological errors, notably the lack of direct comparison with BMB, and the poor performance of PET/CT in evaluating BMI.^21^, ^24^, ^27^, ^28^, ^32^, ^33^, ^34^, ^35^, ^36^, ^37^, ^38^, ^39^ Some of the studies used a different methodological approach, which precludes a straight forward contrast with our results.^40^

St‐Pierre et al., have reported a large series regarding PET/CT in the FL setting.^10^, ^11^ In their work, focused on accuracy, they report a clinically relevant upstage of 16% when considering PET/CT results.^10^ Our data show a superior sensitivity of BMB over PET/CT and, of note, these data do translate into a better accuracy. Focusing on BM evaluation by BMB, only 11 of our patients (3.7%) would have been upstaged to Ann Arbor IV by PET/CT. In the opposite, with PET/CT as the gold standard, 65 (21.7%) would have been upstaged by BMB. Higher percentages of upstaging with the use of PET/CT have been reported by Luminari et al., (7.5%), Le Dortz et al., (18%) and St Pierre et al., (16%). Nakajima et al., focusing on the performance by PET/CT, reported a sensitivity of 69%, and an accuracy of 87%, while in their work BMB showed a sensitivity of 72% and an accuracy of 88%. PET/CT upstaged 24 patients (9.2%) to stage IV.^9^ As we have previously debated, diagnostic performance studies (including ours) comparing BMB vs PET‐CT in this setting inevitably suffer a bias derived from the lack of an independent gold‐standard.^41^, ^42^ Thus, the prognostic value of each technique emerges as a better surrogate marker for clinical utility.

St‐Pierre et al., also reported a survival analysis of their series. They addressed early event free survival (EFS) analyzing extra nodal involvement by PET/CT. In a multivariate analysis with FLIPI‐2 factors, spleen, soft tissue and the pattern of bone involvement, independently predicted a lower EFS, while none of the PET/CT parameters had an independent value for OS prognostication.^11^

Another large series has been reported recently, including 261 patients.^9^ Their results contrast with ours in that PET/CT was the only independent predictor of PFS in multivariate analysis, whereas high FLIPI score and PET/CT predicted OS. This is strikingly different from our multivariate analysis, where PET/CT alone did not achieve independent prognostic value for any clinical outcome. One possible explanation of this contradiction may be that the efficiency of their multivariate model, may be hampered by the bias of collinearity. This bias arises when a factor is partially or totally encompassed by another factor and both of them are included in a multivariate analysis. This overlap of regressors, disrupts the correlation architecture among potentially predictive variables, leading to biased estimations.^43^ Considering BMI by BMB and/or PET/CT as separate factors in a multivariate model that already includes them as part of the FLIPI2 BMI category, may cause a collinearity effect. In our work we tried to overcome this issue, deconstructing the FLIPI2 composite in its foundational factors, considering BMI by means of different measures in four models: BMB+, PET/CT+, “BMB+ and PET/CT+” and “BMB+ or PET/CT+”. Of note, regarding performance analysis, both Nakajima and our conclusion are similar in which the combined “PET/CT and BMB” identify BMI more accurately than either BMB or PET alone.

In the last years, the anticipation of an early relapse has come to the forefront of interest in the setting of FL. For this purpose, several models have been developed to further refine the information obtained in the upfront prognostic indices. Among them, two have reached success and are at present widely used.^32^, ^44^ Though, neither of them have been tested so far with PET/CT. We have addressed this issue considering either of the four definitions of BMI as items of a POD24 model and for this analysis only the combined BMI‐BMB or BMI‐PET/CT was associated with the risk of progression.

We acknowledge that our study design (multicenter series and retrospective nature) confers both strength and weakness and could raise a number of issues. The main limitation of this kind of studies in lymphoma is the lack of a gold standard for the accuracy comparison. Guided repeated biopsies are too invasive. The remission of lesions in end of treatment PET‐CT could be a surrogate marker of baseline involvement of true. Nevertheless, some studies have shown that both lymphomatous and benign/inflammatory bone marrow lesions may demonstrate decrease in uptake at follow‐up.^45^, ^46^ Secondly, relative heterogeneity regarding PET/CT or BMB procedures through different centers, could be argued as a confounding factor. However, we consider that our multicenter approach may be closer to “real life” clinical practice than recent single center reports.^9^, ^10^, ^11^ Thirdly, as in the recent series of Nakajima et al., cases with a diffuse pattern were recorded but not considered as positive BMI.^9^ Diffuse 18FDG uptake is a controversial issue whose real meaning in the setting of FL is unclear: while in some small series it has been related with BMI,^33^ a more recent larger series related this pattern with a high false positive rate.^40^

With a wider perspective, in addition to its added performance and prognosis value, BMB grants both the evaluation of histology (allowing for the detection of either a discordant histology or transformation). When performed with a bone marrow aspirate, it provides suitable material for key ancillary techniques (flow cytometry, cytogenetic and molecular studies).^33^

To conclude, in the upfront workup of FL, PET/CT gives meaningful information regarding nodal and most extranodal areas. In addition, successive studies allow for a clinically relevant response assessment.^15^, ^23^ Our results show that in FL both BMB and PET/CT should be carried out at diagnosis, as their combined assessment provides independent prognostic value in the context of the most widely use clinical scores.

## AUTHOR CONTRIBUTIONS

**Isabel Ródenas Quiñonero:** Conceptualization (lead); data curation (lead); formal analysis (lead); investigation (lead); methodology (lead); resources (lead); supervision (lead); validation (lead); visualization (lead); writing – original draft (lead); writing – review and editing (lead). **Tzu Chen‐Liang:** Conceptualization (lead); data curation (lead); formal analysis (lead); investigation (lead); methodology (lead); supervision (lead); writing – review and editing (equal). **Taida Martín‐Santos:** Resources (equal); supervision (equal); validation (equal); visualization (equal). **Antonio Salar:** Resources (equal); supervision (equal); validation (equal); visualization (equal). **Marta Fernández‐González:** Resources (equal); supervision (equal); validation (equal); visualization (equal). **Carolina Celades:** Resources (equal); supervision (equal); validation (equal); visualization (equal). **José‐Tomás Navarro:** Resources (equal); supervision (equal); validation (equal); visualization (equal). **Martínez Ana Belén:** Resources (equal); supervision (equal); validation (equal); visualization (equal). **Rafael Andreu:** Resources (equal); supervision (equal); validation (equal); visualization (equal). **Aitana Balaguer:** Resources (equal); supervision (equal); validation (equal); visualization (equal). **Alejandro Martín García‐Sancho:** Resources (equal); supervision (equal); validation (equal); visualization (equal). **Mónica Baile:** Resources (equal); supervision (equal); validation (equal); visualization (equal). **Javier López‐Jiménez:** Resources (equal); supervision (equal); validation (equal); visualization (equal). **Juan Marquet‐Palomanes:** Resources (equal); supervision (equal); validation (equal); visualization (equal). **Ana Isabel Teruel:** Resources (equal); supervision (equal); validation (equal); visualization (equal). **María José Terol:** Resources (equal); supervision (equal); validation (equal); visualization (equal). **Carmen Benet:** Resources (equal); supervision (equal); validation (equal); visualization (equal). **Laura Frutos:** Resources (equal); supervision (equal); validation (equal); visualization (equal). **José Luis Navarro:** Resources (equal); supervision (equal); validation (equal); visualization (equal). **Jon Uña:** Resources (equal); supervision (equal); validation (equal); visualization (equal). **Marina Suarez:** Resources (equal); supervision (equal); validation (equal); visualization (equal). **Montserrat Cortes:** Resources (equal); supervision (equal); validation (equal); visualization (equal). **José Contreras:** Resources (equal); supervision (equal); validation (equal); visualization (equal). **Cristina Ruiz:** Resources (equal); supervision (equal); validation (equal); visualization (equal). **Pilar Tamayo:** Resources (equal); supervision (equal); validation (equal); visualization (equal). **Jorge Mucientes:** Resources (equal); supervision (equal); validation (equal); visualization (equal). **Pablo Sopena‐Novales:** Resources (equal); supervision (equal); validation (equal); visualization (equal). **Laura Reguilón‐Gallego:** Conceptualization (equal); data curation (equal); resources (equal); validation (equal); visualization (equal). **José Javier Sánchez‐Blanco:** Resources (equal); supervision (equal); validation (equal); visualization (equal). **Elena Pérez‐Ceballos:** Resources (equal); supervision (equal); validation (equal); visualization (equal). **Andrés Jerez:** Conceptualization (lead); data curation (lead); formal analysis (lead); investigation (lead); methodology (lead); resources (equal); supervision (lead); validation (lead); visualization (lead); writing – original draft (lead). **Francisco José Ortuño:** Conceptualization (lead); investigation (lead); resources (equal); supervision (lead); validation (lead); visualization (lead); writing – original draft (equal); writing – review and editing (equal).

## FUNDING INFORMATION

None.

## CONFLICT OF INTEREST

J.T.N. discloses honoraria from Novartis and Roche; consulting or advisory role in Novartis, Blueprint Medicines and EUSA Pharma; research funding from Gilead and EUSA Pharma. A.S: Roche (Research Funding, Speakers Bureau), Janssen (Consultancy, Speakers Bureau), Gilead (Research Funding), Celgene/BMS (Consultancy), EUSA Pharma (Consultancy), Beigene (Consultancy). A.J. reports a research grant from Gilead and consultancy honoraria from Novartis and Celgene, all outside of the submitted work. The rest of the authors have nothing to disclose.

## Supporting information


Appendix S1

## Data Availability

All data generated during this study are included in this published article and its supplementary information files. The raw data analyzed during the current study available from the corresponding author on reasonable request.
