# Supplementary material for: Accuracy and prognostic impact of FDG PET/CT and biopsy in bone marrow assessment of follicular lymphoma at diagnosis: A Nation‐Wide cohort study
Source: Cancer Med. 2022 Nov 13;12(6):6536–46. doi: 10.1002/cam4.5424 (PMC10067085; doi:10.1002/cam4.5424)
Supplement: Supplementary file 1 — Appendix S1 [file CAM4-12-6536-s001.docx]

**Supplementary Appendix**

**Table 1. Diagnostic performance of PET/CT and BMB for detecting BMI.**

|  | PET/CT+ | PET/CT- | TOTAL |
| --- | --- | --- | --- |
| BMB+ | 37 | 87 | 124 |
| BMB- | 21 | 154 | 175 |
| TOTAL | 58 | 241 | 299 |

Abbreviations: BMB, Bone marrow biopsy; BMI, bone marrow involvement; PET/CT, PET/computed tomography.

**Table 2. Detection of BMI.**

|  | BMI by PET/CT | BMI by BMB |
| --- | --- | --- |
|  | Percentage (95%CI) | |
| Sensitivity (%) | 40.0 (31.6-48.3) | 85.5 (79.4-91.5) |
| NPV (%) | 63.9 (57.6-70.1) | 88.0 (82.9-93.1) |
| Accuracy (%) | 70.9 (65.5-76.2) | 92.8 (89.9-96.0) |

Abbreviations: BMB, Bone marrow biopsy; BMI, bone marrow involvement; NPV: negative predictive value; PET/CT, PET/computed tomography.

**Table 3. Diagnostic performance of PET/CT and BMB for detecting BMI in grade 3 FL patients.**

|  | PET/CT+ | PET/CT- | TOTAL |
| --- | --- | --- | --- |
| BMB+ | 7 | 29 | 36 |
| BMB- | 6 | 43 | 49 |
| TOTAL | 13 | 72 | 85 |

Abbreviations: BMB, Bone marrow biopsy; BMI, bone marrow involvement; PET/CT, PET/computed tomography.

**Table 4. Detection of BMI in grade 3 FL patients.**

|  | BMI by PET/CT | BMI by BMB |
| --- | --- | --- |
|  | Percentage (95%CI) | |
| Sensitivity (%) | 26.5 (13.1-39-9) | 73.4 (60.0-86.8) |
| NPV (%) | 50.0 (37.7-62.2) | 73.4 (60.0-86.8) |
| Accuracy (%) | 57.6 (46.5-68.7) | 84.7 (76.4-92.9) |

Abbreviations: BMB, Bone marrow biopsy; BMI, bone marrow involvement; NPV, negative predictive value; PET/CT, PET/computed tomography.

**Table 5. Univariate analysis of PFS and OS in the whole cohort according to prognostic factors. (Cox proportional hazards model, n=299**).

|  | Univariate model | | | |
| --- | --- | --- | --- | --- |
|  | PFS  p | HR | OS  p | HR |
| Gender, Male vs female | 0.008 | 1.805 (1.164-2-800) | 0.033 | 2.098 (1.062-4.147) |
| Grade, 1,2 vs 3A | 0.601 | 1.134 (0.708-1.816) | 0.422 | 0.742 (0.358-1.536) |
| Score FLIPI, ≤2 vs >2 | 0.011 | 1,746 (1.135-2.687) | 0.001 | 2.997 (1.571-5.714) |
| Score FLIPI 2, ≤2 vs >2 | 0.001 | 2.109 (1.373-3.241) | <0.001 | 4.315 (2.241-8-309) |
| PET/CT BMI, positive vs negative | 0.506 | 1.186 (0.714-1.982) | 0.109 | 1.744 (0.884-3.443) |
| BMB BMI, positive vs negative | 0.012 | 1.730 (1.130-2.650) | 0.035 | 2.011 (1.049-3.856) |
| Combined “PET/CT and BMB” BMI, positive vs negative | 0.283 | 1.383 (0.765-2.501) | 0.285 | 1.562 (0.689-3.541) |
| Combined “PET/CT or BMB” BMI, positive vs negative | 0.017 | 1.701 (1.102-2.627) | 0.011 | 2.460 (1.224-4.944) |
| B2M higher than ULN | <0.001 | 2.275 (1.465-3.543) | 0.008 | 2.483 (1.274-4.840) |
| LoDLIN > 6 cm | 0.002 | 2.031 (1.289-3.199) | 0.074 | 1.894 (0.941-3.813) |
| Hb lower than 120 g/L | 0.025 | 1.733 (1.072-2.804) | 0.004 | 2.569 (1.345-4.908) |
| Age older than 60 y.o. | 0.822 | 1.050 (0.686-1.607) | 0.033 | 2.064 (1.060-4.020) |

Abbreviations: B2M, B2-microglobulin; BMB, Bone marrow biopsy; BMI, bone marrow involvement, FLIPI, Follicular Lymphomas International Prognostic Index; HR, Hazard ratio; Hb, hemoglobin; LoDLIN, longest diameter of the largest involved node; OS, overall survival; PET/CT, PET/computed tomography; PFS, Progression-free survival, ULN, upper limit of normal; y.o, years old.

**Table 6. Univariate analysis of PFS and OS in grade 1-3A FL patients treated with immunochemotherapy according to potential prognostic factors. (Cox proportional hazards model, N=233**).

|  | Univariate model | | | |
| --- | --- | --- | --- | --- |
|  | PFS  p | HR | OS  p | HR |
| Gender, Male vs female | 0.095 | 1.530 (0.929-2,520) | 0.041 | 2.248 (1.034-4.886) |
| Grade, 1,2 vs 3A | 0.478 | 1.220 (0.705-2.111) | 0.373 | 0.679 (0.289-1.591) |
| Score FLIPI, ≤2 vs >2 | 0.003 | 2.103 (1.281-3.453) | 0.001 | 3.684 (1.694-8.013) |
| Score FLIPI 2, ≤2 vs >2 | 0.001 | 2.298 (1.406-3.754) | <0.001 | 4.839 (2.226-10.521) |
| PET/TC BMI, positive vs negative | 0.351 | 1.297 (0.751-2.238) | 0.117 | 1.803 (0.863-3.765) |
| BMB BMI, positive vs negative | 0.009 | 1.952 (1.181-3.228) | 0.008 | 2.981 (1.326-6.699) |
| Combined “PET/CT and BMB” BMI, positive vs negative | 0.293 | 1.402 (0.747-2.632) | 0.168 | 1.808 (0.778-4.202) |
| Combined “PET/CT or BMB“ BMI, positive vs negative | 0.006 | 2.120 (1.239-3.626) | 0.005 | 3.936 (1.511-10.253) |
| B2M higher than ULN | 0.001 | 2.464 (1.467-4.138) | 0.033 | 2.270 (1.067-4.829) |
| LoDLIN > 6 cm | 0.003 | 2.229 (1.324-3.751) | 0.022 | 2.591 (1.148-5.846) |
| Hb lower than 120 g/L | 0.042 | 1.736 (1.019-2.958) | 0.040 | 2.142 (1.036-4.426) |
| Age older than 60 y.o. | 0.958 | 0.987 (0.604-1.611) | 0.372 | 1.385 (0.678-2.830) |

Abbreviations: B2M, B2-microglobulin; BMB, Bone marrow biopsy; BMI, bone marrow involvement, FLIPI, Follicular Lymphomas International Prognostic Index; HR, Hazard ratio; Hb, hemoglobin; LoDLIN, longest diameter of the largest involved node; OS, overall survival; PET/CT, PET/computed tomography; PFS, Progression-free survival, ULN, upper limit of normal; y.o, years old.

**Table 7. Prognostic value for PFS and OS of variables considered within the FLIPI2 score in the cohort of patients treated with an intensive regimen. Two multivariate models were performed for PFS. Three multivariate models were performed for OS (Cox proportional hazards model, n=233).**

|  | Multivariate model considering BMB for BMI | | | | Multivariate model considering combined “PET-CT or BMB” for BMI | | | | Multivariate model considering PET-CT for BMI | |
| --- | --- | --- | --- | --- | --- | --- | --- | --- | --- | --- |
|  | PFS  p | HR (95% CI) | OS  p | HR (95% CI) | PFS  p | HR (95% CI) | OS  p | HR (95% CI) | OS  p | HR (95% CI) |
| B2M higher than ULN | 0.081 | 1.694 (0.937-3.063) | 0.505 | 1.371 (0.543-3.460) | 0.131 | 1.590 (0.871-2.902) | 0.468 | 1.407 (0.560-3.538) | 0.379 | 1.515 (0.601-3.817) |
| LoDLIN >6 cm | 0.011 | 2.027 (1.174-3.499) | 0.064 | 2.242 (0.955-5.265) | 0.018 | 1.939 (1.119-3.361) | 0.050 | 2.309 (1.000-5.334) | 0.056 | 2.281 (0.978-5.317) |
| Hb lower than 120 g/L | 0.635 | 1.168 (0.616-2.213) | 0.675 | 1.240 (0.455-3.380) | 0.540 | 1.220 (0.646-2.304) | 0.723 | 1.191 (0.452-3.135) | 0.806 | 1.129 (0.428-2.978) |
| Age older than 60 y.o. | 0.433 | 0.804 (0.466-1.386) | 0.300 | 1.574 (0.668-3.709) | 0.450 | 0.810 (0.470-1.398) | 0.244 | 1.642 (0.713-3.782) | 0.439 | 1.392 (0.602-3.218) |
| BMI by:  PET/TC  BMB  Combined “PET/CT and BMB positive”  Combined “PET/CT or BMB positive” | NE  0.024  NE  NE | NE  1.903 (1.090-3.322)  NE  NE | NE  0.013  NE  NE | NE  3.286 (1.280-8.436)  NE  NE | NE  NE  NE  0.009 | NE  NE  NE  2.247 (1.222-4.132) | NE  NE  NE  0.013 | NE  NE  NE  3.940 (1.333-11.651) | 0.313  NE  NE  NE | 1.545 (0.664-3.593)  NE  NE  NE |

Abbreviations: B2M, B2-microglobulin; BMB, Bone marrow biopsy; BMI, bone marrow involvement; HR, Hazard ratio; Hb, hemoglobin; LoDLIN, longest diameter of the largest involved node; NE, not entered; OS, overall survival; PET/CT, PET/computed tomography; PFS, Progression-free survival, ULN, upper limit of normal; y.o, years old.

**Table 8. Univariate analysis for PFS of BMI categories in low and intermediate *PRIMA-PI score patients.* (Cox proportional hazards model, n=171**).

|  | Univariate model | |
| --- | --- | --- |
|  | PFS  p | HR |
| PET/TC BMI, positive vs negative | 0.332 | 1.432 (0.693-2.961) |
| BMB BMI, positive vs negative | 0.029 | 2.068 (1.078-3.967) |
| Combined “PET/CT and BMB” BMI, positive vs negative | 0.119 | 1.932 (0.845-4.419) |
| Combined “PET/CT or BMB“ BMI, positive vs negative | 0.044 | 2.003 (1.019-3.938) |

Abbreviations BMB, Bone marrow biopsy; BMI, bone marrow involvement; HR, Hazard ratio; OS, overall survival; PET/CT, PET/computed tomography; PFS, Progression-free survival.

**Table 9. Comparison of baseline characteristics between “PET/CT+ and BMB–“ vs “PET/CT+ and BMB +“.**

| Characteristics | 1. PET/CT+ /BMB – (n=21) | 1. PET/CT + / BMB+ (n=37) | p (A vs B) |
| --- | --- | --- | --- |
| Age at diagnosis (y.o), mean (±SD) | 56.7±13.2 | 61±12.1 | 0.226 |
| Female/male, (%) | 57/43 | 59/41 | 0.956 |
| WBC (×10E9/L), mean (±SD) | 11.1±20.6 | 8.2±5.7 | 0.531 |
| Hb (g/dL), mean (±SD) | 13.1±2.1 | 12.7±2.1 | 0.519 |
| Platelets (×10E9/L), mean (±SD) | 245.6±93.7 | 205.5±87.1 | 0.114 |
| B2microglobulin [ULN], (%) | 40/60 | 61/39 | 0.116 |
| LoDLIN >6 cm, (%) | 32/68 | 42/58 | 0.464 |
| LDH U/L, mean (±SD) | 291.1±117.2 | 315.1±125.3 | 0.484 |
| B symptoms, (%) | 14/86 | 34/66 | 0.112 |
| Splenomegaly,(%) | 33/67 | 49/51 | 0.251 |

Abbreviations: B2M, B2-microglobulin; BMB, Bone marrow biopsy; Hb, hemoglobin; LoDLIN, longest diameter of the largest involved node; PET/CT, PET/computed tomography; ULN, upper limit of normal; WBC, white blood cells; y.o, years.
